# Immunology and the Central Nervous System

**DOI:** 10.1155/2013/512684

**Published:** 2013-12-31

**Authors:** Carlos Barcia, James Curtin, Jeffrey Zirger, Daniel Larocque

**Affiliations:** ^1^Department of Biochemistry and Molecular Biology, Institute of Neuroscience and School of Medicine, Universitat Autonoma de Barcelona, Campus de Bellaterra, Cerdanyola del Valles, Barcelona, Spain; ^2^School of Food Science and Environmental Health, Dublin Institute of Technology, Dublin, Ireland; ^3^Centers for Disease Control and Prevention (CDC), Atlanta, GA, USA; ^4^Immunotherapeutics BU, GlaxoSmithKline Vaccines, Laval, QC, Canada

We are pleased to announce the publication of this special issue in the journal “Clinical and Developmental Immunology.” We are happy to perceive the growing interest, from a wide range of scientists, regarding the peculiar intercommunication between the immune system and the central nervous system (CNS). We have finally reached a balanced compilation of papers that we collect in this special issue highlighting recent fundamental advances in our understanding of brain immunology with an emphasis on new therapeutic targets covering such emerging topics as chemical suppression of glial activation, inflammation following acute demylination, Notch signalling as a potential therapeutic target in EAE and a link between neuroinflammatory signaling and reproduction.

The idea that the CNS is an immune-privileged site is gradually vanishing. However, increasing evidence shows that the relation between the CNS and the immune system is special and in many aspects, different from the rest of the organs and tissues [1].

One of the peculiarities of the CNS is the presence of glial cells, which are the initial responders to brain injuries and degenerative processes [2]. Glial cells, especially microglia, get locally activated in the damaged brain areas and are able to induce the recruitment of scavenger blood cells, such as monocytes and lymphocytes to injured sites, contributing to the inflammatory reaction [3, 4]. In our special issue, R. A. Taylor and L. H. Sansing, describe, in a comprehensive review, the role of microglial cells in ischemic stroke and intracerebral hemorrhage. Importantly, authors highlight the distinct phenotypes of microglia, M1-inflammatory, and M2-repairing microglia, which express different surface molecules and releasing factors. They propose that understanding the mechanism of this switching phenotype will be crucial for future therapeutic purposes. In line with this, E. Assi et al., review the recent literature about the role of microglia in inflammatory signaling cascades in brain pathology, but focusing their paper on the role of sphingolipids in the inflammatory reaction, which is proposed as a potential target to control glial-mediated neuroinflammation.

In this context, it is becoming more evident that glial activation is a critical event that should be targeted to avert inflammation in the CNS. B. Rocamonde et al. describe, in an original study, using a rat model of brain injury, that the reduction of glial activation by lipoic acid underlies the restorative effects in the brain. F. Cloutier et al. suggest in an interesting paper that the role of microglia and astrocytes during spinal cord injury and repair may be different depending on the scenario of CNS damage. Authors report, using a rat model of acute demyelination in dorsal funiculus, how immunological using anti GalC demyelination triggers macrophage/microglial cells activation in comparison of a stab injury. Interestingly, in their model of axon regeneration, the participation of astrocytes is limited, whereas microglia and infiltrated macrophages have a prominent role, which emphasize the potential of targeting microglial cells for therapeutic purposes.

From this perspective, papers on CNS autoimmunity, MS and MS' experimental models have largely contributed to our special issue. We have compiled extensive reviews and original papers about the initial immune-pathogenesis of MS as well as the immunology and oxidative stress underlying the disease. R. Bassil et al. provide a comprehensive reassessment on the important function of Notch signaling in the activation of T helper cells in experimental autoimmune encephalitis (EAE). They propose targeting Notch signaling as a potential strategy for MS therapy, but they also report the limitations of this approach due to the wide range of functions of Notch signaling, suggesting interesting new research avenues. Hernández-N. Y. Pedro et al. discuss the innate immune responses occurring during the immune-pathogenesis of MS, which are hypothesized as a critical trigger of the chronic inflammatory response. In line with this, G. G. Ortiz et al. describe that oxidative stress and inflammation are important elements in the self-perpetuation inflammatory/immune cycle of MS. These pathways may contain targets that could be promising to avert the inflammatory responses in MS. Concluding this MS section from a practical point of view, S. F. Gonçalves Zorzella-Pezavento et al contribute to settle down the potential controversy regarding the tuberculosis (TB) vaccination protocols, suggesting that TB vaccination does not trigger or worsen EAE pathology.

Immunotherapy is also an important topic covered by this special issue. In the CNS, T-lymphocytes infiltrate into the inflamed brain parenchyma and the manipulation of different T-cell subpopulations may have beneficial effect on neurodegenerative disorders and diverse brain injuries. T cell infiltration is observed in many neurodegenerative diseases including Parkinson's and Alzheimer's disease and we are getting to know more aspects on how T-cells may affect neurodegeneration [5, 6]. Importantly, immunotherapy based on targeting T cells (T_regs_ and Th17 cells) for autoimmunity disorders and cancer is becoming a rising field of investigation [7]. However, the precise lymphocyte function in the damaged brain parenchyma is still poorly understood. S. Chen et al. propose, in a completed review, that the modulation of T_regs_ may have a favorable outcome for stroke patients. This is based on the fact that T_regs_ downregulates the excessive brain immune-reaction, which could be favorable for tissue restoration. In line with this, M. S. Zaborowski and Michalak describe how the appearance of paraneoplastic neurological syndromes may be linked with the certain antitumor responses triggered by T cells. Thus, authors suggest that the manipulation of specific subpopulation of T cells, pondering the balance between T_regs_ and CTLs through immunotherapy, could be beneficial for these neurological disorders.

In this context of immunotherapy, the search for antigens that may trigger autoimmune responses in the brain is a crucial field of research. The review by A. Seppänen proposes collagen XVII as a candidate antigen. Autoantibodies against different domains of collagen XVII are found elevated in serum of patients within a wide range of neurological disorders as well as in skin autoimmune disorders, suggesting that an autoimmune response may be the common trigger of those disorders. On the other hand, increasing evidence indicates that immune system dysfunction may also be underlying a number of psychiatric disorders. K. Pathmanandavel et al. hypothesize in their thought-provoking review that some neuronal autoantibodies may be linked with psychiatric symptoms and could open new immunomodulatory approaches for neuropsychiatric disorders in the near future.

We scarcely know the factors that trigger CNS diseases. It is thought that genetic and environmental factors, which include the exposure to chemical compounds or particular pathogens, may contribute to the appearance of these disorders. T. T. Win-Shwe et al. discuss an interesting point regarding the immune-related inflammation that environmental volatile compounds may induce. The review hypothesizes how environmental volatile elements may affect respiratory and immune system, having neurological consequences for the population. On the other hand, T. Hautala et al. report in our special issue how viruses, in this case *Puumala* virus, a northern European type of *Hantavirus*, are important entities that may seriously affect the CNS. Other pathogen infections, such as *Trypanosoma brucei*, which causes sleeping sickness, may also cause severe brain damage. Interestingly, D. N. Maranga et al., members of a research group based in Kenya, present in our special issue a research study performed in few monkeys showing that the increase of the proinflammatory cytokine IL-6 in spinal fluid may be a reliable marker to manage human African trypanosomiasis in areas where the diagnostic tools are very limited and derived neurological consequences are frequent. Lastly, A. P. Herman et al. describe how the inflammatory-mediated release of cytokines, such as IL-1*β*, may have consequences in the hypothalamic neurons affecting the reproductive system. This highlights that it is also important to consider the effect that neuroimmune interactions and neuroinflammation may have in many other systems.

We hope this collection of papers is helpful for readers to understand better the peculiar relationship between the immune system and CNS and may be a stimulus to continue the research on this complex field.

*Carlos Barcia* *James Curtin* *Jeffrey Zirger* *Daniel Larocque*

## References

[1] Ransohoff RM, Brown MA (2012). Innate immunity in the central nervous system. *The Journal of Clinical Investigation*.

[2] Ilieva H, Polymenidou M, Cleveland DW (2009). Non-cell autonomous toxicity in neurodegenerative disorders: ALS and beyond. *The Journal of Cell Biology*.

[3] Engelhardt B, Ransohoff RM (2012). Capture, crawl, cross: the T cell code to breach the blood-brain barriers. *Trends in Immunology*.

[4] Wraith DC, Nicholson LB (2012). The adaptive immune system in diseases of the central nervous system. *The Journal of Clinical Investigation*.

[5] Appel SH, Beers DR, Henkel JS (2010). T cell-microglial dialogue in Parkinson’s disease and amyotrophic lateral sclerosis: are we listening?. *Trends in Immunology*.

[6] Goverman J (2009). Autoimmune T cell responses in the central nervous system. *Nature Reviews Immunology*.

[7] von Boehmer H, Daniel C (2013). Therapeutic opportunities for manipulating T(Reg) cells in autoimmunity and cancer. *Nature Reviews Drug Discovery*.

